# The contingency effects of abusive supervision of coworker on helping behaviors: the relationships among the perpetrator, the victim, and the third party

**DOI:** 10.3389/fpsyg.2026.1755436

**Published:** 2026-02-17

**Authors:** Meng Sun, Liu-Tian Luo, Duan-Ni Zhao

**Affiliations:** 1 Faculty of Hospitality and Tourism Management, Macau University of Science and Technology, Taipa, Macao SAR, China; 2 Shanwei Institute of Technology, Shanwei, Guangdong, China

**Keywords:** abusive supervision of coworker, deep-level similarity, deontic justice theory, helping behaviors, moral attentiveness, perceived supervisor support, social identity theory

## Abstract

**Introduction:**

Abusive supervision, characterized by hostile verbal and non-verbal behaviors by supervisors, has been recognized as a significant stressor in the workplace. Although much research has focused on the victim’s and perpetrator’s perspectives, little attention has been given to third-party witnesses and their reactions to abusive supervision, particularly in hospitality contexts. This study investigates the contingent effects of coworker abusive supervision on third-party helping behaviors, focusing on the tripartite relationship between the perpetrator, victim, and witness.

**Methods:**

We conducted a field study with 500 employees from various departments in seven four- and five-star hotels in Guangdong, China. Using a two-wave time-lagged survey design, participants completed measures on abusive supervision, deep-level similarity, perceived supervisor support, moral attentiveness, and helping behaviors. Structural equation modeling (SEM) was employed to test the hypothesized relationships and moderating effects.

**Results:**

The study found that the relationship between coworker abusive supervision and third-party helping behaviors was moderated by three factors: deep-level similarity with the victim, perceived supervisor support, and moral attentiveness. Specifically, helping behavior was more likely to decrease when similarity with the victim was low, supervisor support was high, and moral attentiveness was low. The results underline the complex nature of third-party responses to abusive supervision.

**Discussion:**

This research extends existing literature by highlighting the conditional factors that influence whether third-party witnesses decide to help an abused coworker. The findings suggest that interventions in abusive supervision cases should consider not only the relationship between the victim and perpetrator but also the personal characteristics and contextual factors influencing the witnesses. The study provides valuable insights into how workplace dynamics and moral concerns shape responses to supervisory abuse.

## Introduction

Abusive supervision is defined as subordinates’ perceptions of continuous hostile verbal and nonverbal behaviors perpetrated by employees’ immediate supervisors and is one of the main stressors in the workplace (Tepper, 2000). Research over the past two decades has suggested that this destructive leadership style not only deteriorates the subordinate’s (victim’s) outcomes, but also backfires, damaging the supervisor’s (perpetrator’s) ability to function at work. On the one hand, experiencing abusive supervision is a salient negative workplace event that often results in extensive, undesirable consequences for the subordinate, such as diminished job performance and decreased organizational commitment (Zhang and Liao, 2015), increased psychological distress (Tepper, 2000), coworker-directed deviance behaviors, and family undermining behaviors (Li and Xu, 2025; Pradhan and Gupta, 2021). On the other hand, abusive supervisors may the recipients of retaliatory actions by subordinates who may say something harmful to the supervisor or gossip about them (Mitchell and Ambrose, 2007). Managers perceive abusive supervisors as less effective and will give them lower ratings in terms of in-role performance (Ambrose and Ganegoda, 2020). Supervisors may also experience a sense of guilt and a loss of moral credit after performing abusive supervisory behaviors (Liao et al., 2018). Until now, the extant literature has provided a deep level of understanding of the impacts of abusive supervision, from the perspectives of both the victim and the perpetrator.

The hospitality sector provides a particularly relevant context for examining coworker abusive supervision because frontline service work is highly interdependent and often relies on discretionary cooperation among employees to maintain service quality and respond to customer demands (Zhu et al., 2024). In such settings, helping behaviors toward coworkers are not only socially desirable but also instrumental for daily operations, service recovery, and team coordination (Paşamehmetoğlu et al., 2022; Zhu et al., 2024). At the same time, hotels often feature hierarchical structures, intense workload cycles, and high turnover, which may increase employees’ exposure to abusive supervisory behaviors and amplify their spillover effects beyond direct targets (Ghosh et al., 2024; Üngüren et al., 2024). Accordingly, understanding when witnesses choose to help or withdraw support from an abused coworker is especially important for sustaining service functioning in hospitality organizations (Ghosh et al., 2024; Zhu et al., 2024).

Although abusive supervision occurs in a dyadic relationship, an ecological perspective suggests that employee behaviors largely depend on contextual factors in the workplace (Dunn et al., 1994). In the same vein, social learning theory asserts that employees learn what constitutes acceptable behavior at work by observing how the supervisors treat other employees (Bandura, 1977). This infers the possibility that witnessing a coworker being abused by the supervisor elicits a reaction from a third-party employee, even when the witness does not experience the same treatment. Accordingly, given that the effects of abusive supervision on victim-related and perpetrator-related consequences have received the great majority of the research attention, a small but growing stream of research has focused on how abusive supervision of a coworker affects third-party employees. Recent studies published in Frontiers in Psychology have similarly emphasized the critical role of bystanders in responding to workplace mistreatment and the potential risks associated with intervention, which aligns with the third-party focus of the present study (Borthakur et al., 2026; Chen et al., 2022). In addition, Xu et al. (2020) found that employees who witness the abuse experience schadenfreude and engage in more destructive behaviors toward the abused coworker, especially when the interpersonal rivalry is high. To the contrary, Priesemuth and Schminke (2019) found that employees who witness the abuse feel angry and display more protective behaviors toward the abused employee, especially when the working environment is fair. The consensus of this stream of research is that third-party employees have different emotional and behavioral reactions to a supervisor’s abuse of a coworker, depending on their subjective appraisal of the event. Nevertheless, most of the research has considered only a single moderator when investigating how the abusive supervision of a coworker affects a third-party employee’s behavioral reaction.

Given the contextual complexity of the abusive supervision of a coworker—in which a perpetrator, a victim and an observer are all involved - we propose that the third-party employee’s reaction will be influenced by the appraisals of dependent others and the self. As such, we develop an integrated framework to explore how the relationship between the abusive supervision of a coworker and helping behaviors toward the victim is contingent upon three moderators: deep similarity with the coworker (victim-related factor), perceived supervisor support (perpetrator-related factor), and moral attentiveness (witness-related factor). We include deep similarity with the coworker and perceived supervisor support because social identity theory (Ashforth and Mael, 1989) asserts that individuals often define themselves relative to people in other categories and engage in behaviors that benefit the members of their group Hence, whether third party employees help the abused coworker depends on the target (either the perpetrator or the victim) with whom they identify. Moreover, we include moral attentiveness because deontic justice theory (Folger and Cropanzano, 2001) proposes that mistreating others may motivate individuals to take action to restore fairness for the victim, particularly when they exhibit a heightened awareness of moral concerns. Therefore, this study primarily aims to explore the conditions under which third-party employees, who witness abusive supervision, choose to help or not help the abused coworker. Specifically, the study examines how deep-level similarity with the victim, perceived supervisor support, and the witness’s moral attentiveness moderate this relationship. We expect the negative relationship between abusive supervision of a coworker and helping behaviors to be more pronounced under specific conditions, such as when deep-level similarity with the coworker is low, perceived supervisor support is high, and moral attentiveness is low. In the opposite conditions, we also anticipate the potential for an increase in helping behaviors.

Our research has several theoretical implications. First, by considering multiple moderators simultaneously, we illustrate the decision-making process through which third-party employees respond to abusive supervision directed at a coworker. This integrated approach suggests that witnesses’ reactions are shaped by their perceptions of both the perpetrator and the victim, as well as by moral considerations. Second, this study identifies key boundary conditions in a social ecological context, namely deep-level similarity with the victim, perceived supervisor support, and moral attentiveness, which help clarify when third-party helping toward an abused coworker is more likely to be constrained or reduced. In doing so, this study extends prior research on workplace helping by highlighting how interpersonal dynamics and moral attentiveness jointly shape behavioral responses to observed mistreatment. Third, we contribute to hospitality management research by examining how witnesses interpret supervisors’ paradoxical cues when constructive and destructive behaviors coexist, offering insight into how such mixed signals may shape employees’ behavioral responses in situations involving coworker abuse.

## Theoretical background and hypothesis development

### To help or not to help: two perspectives

The literature on abusive supervision has documented how experiencing abusive supervision may lessen individual interpersonal citizenship behaviors (Liu et al., 2024). Prior research has shown that victimized employees may reproduce the abusive supervision in their interactions with others because they have learned that a positive and ethical exchange relationship is not appreciated. Withholding prosocial and proactive behaviors at work is an alternative way to respond to the negative supervisory treatment and restore a means of autonomy and fairness. However, the relationship between the abusive supervision of a coworker and the witnesses’ helping behaviors toward the coworker is unclear. Importantly, abusive supervision of a coworker represents a third party context in which employees observe a coworker being mistreated rather than directly experiencing abuse themselves. Although witnesses are not the immediate targets, prior research indicates that observing coworker abuse can still elicit strong emotional and behavioral reactions, including moral outrage, anger, disengagement, and even malicious responses toward the victim (Mitchell et al., 2015; Priesemuth and Schminke, 2019; Xu et al., 2020; Yu et al., 2022). In this third party context, helping behavior becomes a discretionary decision that often involves a trade-off between moral considerations (e.g., restoring fairness for an unjustly treated coworker) and self-protective concerns (e.g., avoiding retaliation or relationship costs in hierarchical settings) (Priesemuth and Schminke, 2019; Yu et al., 2022). Therefore, it is theoretically important to clarify the conditions under which witnesses will help versus withhold help when coworker abusive supervision occurs.

The deontic justice theory suggests that when witnessing others being treated unethically, individuals may experience moral outrage and feel obliged to fight for what they believe is fair and just, even when this mistreatment does not have a direct implication to themselves (Folger and Cropanzano, 2001). Accordingly, research has evidenced that employees tend to engage in more helping behaviors toward the victim when witnessing others being mistreated at work. Nevertheless, deontic concerns may not always translate into overt helping behaviors in coworker abusive supervision episodes (Priesemuth and Schminke, 2019; Yu et al., 2022). Helping the victim can be perceived as a visible act of siding against the supervisor, which may expose bystanders to interpersonal costs and potential retaliation, particularly in hierarchical work settings (Park et al., 2018; Wei et al., 2023). Therefore, even when witnesses morally disapprove of the mistreatment, they may maintain the status quo or withhold discretionary assistance rather than proactively increasing help (Priesemuth and Schminke, 2019; Wei et al., 2023; Yu et al., 2022).

Beyond these situational constraints, other researchers have argued that an individual’s justice judgment is often biased by their subjective evaluation of the target (Blader, 2007). Social identity theory proposes that individuals are likely to categorize people into in-groups and out-groups based on previous interactional experiences, and will become identified with a certain target, which further impacts their work behaviors (Tajfel et al., 1979). When identification with the supervisor is high, third-party employees are more likely to rationalize supervisory abusive behaviors and consider the abused coworker as deserving the mistreatment (Chen and Liu, 2019). The same logic can be applied to the situation when identification with coworker is low. Research has indicated that third party employees do not support the abused coworker if a highly competitive relationship exists between themselves and the abused coworker (Chen et al., 2021), or when they negatively evaluate the abused coworker (Mitchell et al., 2014). These findings offer explicit evidence that, in some cases, a negative relationship exists between the abusive supervision of a coworker and helping behaviors toward that coworker.

To integrate these relevant but piecemeal studies and develop a comprehensive picture of this phenomenon, we assume that the relationship between the abusive supervision of a coworker and helping behaviors toward the coworker is contingent upon three moderators: similarity, perceived supervisor support, and moral attentiveness. We base this on deontic justice theory and social identity theory. In the following sections, we discuss the theory behind our three proposed moderating hypotheses.

### Moderating effect of similarity between witness and abused coworker

Deep-level similarity involves a subjective assessment of the congruence of personal attitudes, opinions, values and beliefs with those of others (Harrison et al., 2002). It pertains to the core elements of an individual’s identity, which, although less visible, are essential for profound interpersonal connections. Theoretically, there are types of similarity, however, the current study focuses on deep-level similarity rather than surface-level similarity for several reasons. First, deep-level similarity wields more influence than does surface-level similarity, as the characteristics of the former enable more accurate inferences and a deeper understanding of others. In contrast, surface-level similarity provides less direct insight into an individual’s core self, thereby creating greater potential for stereotypes and biases (Żerebecki et al., 2024). Second, deep-level similarity has been shown to have a more consistent and enduring impact compared to surface-level similarity (Li et al., 2022). As research suggests, coworkers who spend extended periods together exchange more information, leading to richer and more effective interactions (Huang et al., 2024).

In our research context, we argue that the likelihood of third parties offering help to coworkers who have been abused by a supervisor is contingent upon the deep-level similarity between the coworkers and third parties. Individuals define themselves in relation to the groups to which they belong, and tend to view members of similar groups more favorably than those of dissimilar groups (Goldberg, 2003). Individuals are generally more attracted to those who share similar personality traits, attitudes and values (Byrne, 1971), which may strengthen mutual identification and psychological closeness among coworkers. When deep-level similarity with the victim is high, witnesses may be more likely to remain supportive of the abused coworker and sustain their willingness to help during negative workplace events.

Prior research suggests that third-party employees do not always respond to observed abusive supervision with supportive actions; instead, some witnesses may avoid involvement or withdraw from the situation altogether, particularly when intervention is perceived as risky (Priesemuth and Schminke, 2019). In contrast, when deep-level similarity is low, the abused coworker may be perceived as a relatively distant other, and witnesses may feel less obligation to invest in discretionary assistance. Under such circumstances, third-party employees may be more likely to refrain from offering additional help when the coworker is abused by the supervisor, particularly because such involvement may be interpreted as challenging the supervisor and may expose the witness to interpersonal or retaliatory risks (Jennings et al., 2024). Therefore, we expect deep-level similarity to shape whether third-party employees respond to coworker abusive supervision with supportive actions or with behavioral withdrawal. Specifically, when deep-level similarity is high, witnesses may be more inclined to maintain or offer support to the abused coworker, whereas when deep-level similarity is low, witnesses may be more likely to withhold discretionary assistance. Based on the above arguments, we formulate the first hypothesis.

*Hypothesis 1*: Deep-level similarity with the abused coworker moderates the relationship between abusive supervision of coworker and helping behaviors toward the coworker, such that the negative association is more likely to emerge when deep-level similarity is low rather than high.

### Moderating effect of witness’s perceived supervisor support

Previous studies have separately examined abusive supervision and supervisor support. We posit that perceived supervisor support and perceptions of abusive supervision can coexist simultaneously. Leaders who engage in abusive supervision behaviors may also demonstrate supportive behaviors concurrently (Duffy et al., 2002). There are several evidence to support this notion. First, Chénard-Poirier et al. (2021) has shown that constructive supervisor behaviors (e.g., transformational leadership) and destructive supervisor behaviors (e.g., petty tyranny) can coexist and even interact with one another. That is because that leader’s constructive or destructive behaviors would be elicited by specific work events or situations. For example, when supervisors work experience high work stress on a given day, they tend to display more constructive leadership. Conversely, when supervisors experience low work stress on another day, they may display more destructive leadership. Thus, supervisors’ behavior toward employees is not always consistent (Lian et al., 2012) and constructive behaviors (e.g., supervisor support), making their coexistence reasonable.

Second, Agarwal (2019) study also demonstrated that supervisors may exhibit the abusive supervision toward the employees with whom high leader-member exchange. That is, when leaders’ self-regulation resources are depleted, they may be unable to suppress negative emotions, resulting in abusive supervisory behaviors, even in the context of a good relationship with subordinates (Lian et al., 2012). Therefore, abusive supervision and perceived supervisor support can coexist.

To counter the previous arguments, some research has asserted that when coworkers are abused by their supervisors, third parties may choose not to provide the help. For example, supervisor’s coercive power may influence employee reactions. Third parties believe that helping the victim may ruin their relationship with the supervisor, because witness’s helping behaviors would be regarded as a form of disagreement with their behavior by supervisor. This further creates misunderstandings and conflicts between the witness and the supervisor and has a negative impact on witness’s career development. In addition, to prevent themselves from becoming the next target of the same misconduct, witnesses are likely to keep silent and try to not get involved in the event (Afshan et al., 2022). Hence, this study joins this stream of research, focusing on a perpetrator-related factor, perceived supervisor support, as a moderating variable as we examine situations in which third parties do not help their coworkers who are experiencing abusive supervision.

Perceived supervisor support refers to employees’ overall perception of how much their superiors value their contributions and care about their wellbeing (Eisenberger et al., 2002). Employees who perceive a higher level of supervisor support gain access to more work resources, such as job opportunities and promotions, placing them in a favorable position relative to their coworkers. As a result, to ensure the continuity of resource availability, they must demonstrate loyalty to their superiors in all aspects of their work (Garg and Dhar, 2016). This kind of exchange may also facilitate the development of mutual trust and understanding, which can prompt them to rationalize the supervisors’ actions. Such rationalization may lead witnesses to downplay the severity of the supervisor’s behavior and prioritize relationship maintenance, making them less willing to engage in actions that could be interpreted as dissent. Thus, when witnessing the abusive supervision of a coworker is a single episodic event, the witness may choose to interpret the supervisor’s behavior and motivation based on previous interactional experiences and the existing trust between them, and thus believe in the supervisor’s good intention (Chen and Liu, 2019). As a result, the witness may reduce the helping behaviors toward the abused coworker.

Consistent with this notion, the literature on abusive supervision has demonstrated that employees who have a closer relationship with the supervisor are more likely to interpret that supervisor’s abusive supervision in a positive and constructive way (e.g., as an attempt to motivate their coworkers to achieve higher performance) (Yu and Duffy, 2021) or forgive supervisor’s misconduct (Yang et al., 2020). This is more likely to be the case when the core individual is not a victim but a witness. Based on the above discussion, we formulate the following hypothesis.

*Hypothesis 2*: Perceived supervisor support moderates the relationship between abusive supervision of coworker and helping behaviors toward the coworker, such that the negative association is more likely to emerge when perceived supervisor support is high rather than low.

### Moderating effect of witness’ moral attentiveness

To external factors, individual dispositions also guide behavioral responses in the workplace. Abusive supervision is widely regarded as a moral transgression that violates fundamental norms (e.g., care and nonmaleficence) and may threaten observers’ moral self-concept. Moral attentiveness, defined as “the degree to which individuals discern and contemplate moral aspects within their experiences across time” (Reynolds, 2008), has therefore received substantial attention in abusive supervision research. Prior work has examined moral attentiveness from multiple perspectives. For instance, abusive supervisors with higher levels of moral attentiveness are more likely to reexamine their wrongdoing and reflect on the harm inflicted on subordinates (Liao et al., 2018). Relatedly, Pan et al. (2023) suggested that moral attentiveness can shape how individuals interpret and respond to unethical managerial behaviors. Extending this stream of research, we consider the witness’s moral attentiveness as a boundary condition that shapes helping responses toward an abused coworker.

**Figure 1:**
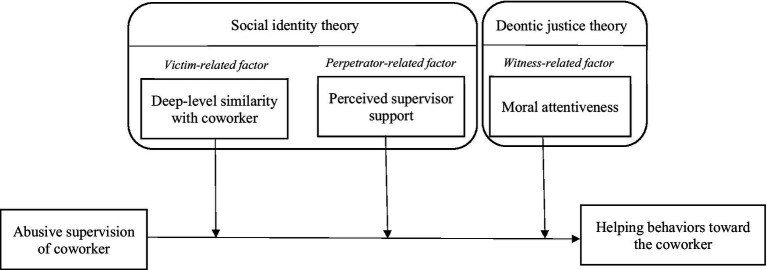
Research model.

Moral attentiveness includes both perceptual and reflective components (Reynolds, 2008). Perceptual moral attentiveness captures the extent to which individuals chronically notice moral elements in everyday experiences, whereas reflective moral attentiveness captures the extent to which individuals deliberately consider moral implications and evaluate alternative actions (Reynolds, 2008). In principle, witnesses with higher moral attentiveness may be more sensitive to the unfairness of coworker mistreatment and may experience stronger moral discomfort when observing abusive supervision (Yu et al., 2022). However, even when individuals attend to the moral aspects of an event, behavioral responses may still be shaped by contextual constraints and self-regulatory processes, such that moral considerations may not necessarily translate into overt action (Wang and Xiao, 2022). In abusive supervision episodes, power imbalance and concerns about retaliation may discourage witnesses from engaging in visible supportive behaviors (Park et al., 2018; Wei et al., 2023; Yu et al., 2022). Thus, even when witnesses recognize the moral problem, they may refrain from helping to protect themselves from potential negative consequences (Wei et al., 2023; Yu et al., 2022; Figure 1).

Accordingly, we propose that moral attentiveness primarily influences whether witnesses withdraw help under coworker abusive supervision rather than guaranteeing increased helping. Specifically, when moral attentiveness is low, witnesses may be less likely to perceive the moral severity of the event and less likely to deliberate about appropriate responses, making them more prone to reduce discretionary assistance toward the abused coworker. In contrast, when moral attentiveness is high, witnesses may be more attentive to the moral implications of the mistreatment, which can buffer against behavioral withdrawal, even if it does not necessarily prompt active intervention. Therefore, we propose the following hypothesis.

*Hypothesis 3*: Moral attentiveness moderates the relationship between abusive supervision of coworker and helping behaviors toward the coworker, such that the negative association is more likely to emerge when moral attentiveness is low rather than high.

## Method

### Participants and procedure

To reduce common method variance (CMV), this study employed a two-wave time-lagged survey design with a four-week interval between data collections. This design helps mitigate respondents’ tendency to provide consistent answers across measures collected at the same time by introducing temporal separation between predictors and outcomes (Podsakoff et al., 2003). We adopted a two-wave design because our focal variables are relatively stable individual perceptions and dispositions, whereas helping behavior reflects subsequent behavioral responses. A four-week interval was chosen to provide sufficient temporal separation to reduce consistency motives and recall of earlier responses, while remaining short enough to ensure that the focal work context and coworker relations were unlikely to change substantially during the study period. The sample was drawn from seven four- and five-star hotels located in Guangdong Province, China. These hotels were selected using a purposive sampling approach with convenience access. Specifically, we initially contacted 12 four- and five-star hotels in Guangdong Province through the authors’ professional networks and alumni/industry contacts, and seven hotels agreed to participate after internal approval by their human resources departments. We focused on four- and five-star hotels because they typically have standardized supervisory structures, formal HR systems, and high levels of task interdependence among frontline employees, which provide an appropriate context for examining coworker abusive supervision and subsequent behavioral reactions. To reduce the risk that results were driven by a single organization, data were collected from multiple hotels and across several operational departments (e.g., front office, food and beverage, and housekeeping).

Before the data collection, we contacted the human resources departments of all participating hotels to obtain permission and coordinate the survey process. Research assistants visited each hotel, met with department managers (including those from the front office, food and beverage, housekeeping, and other departments), and explained the study purpose and confidentiality procedures. Participation was voluntary and anonymous. With the assistance of HR staff and department managers, questionnaires were distributed on-site to eligible frontline employees during work breaks, and participants returned the completed surveys in sealed envelopes to collection boxes placed in the HR office.

Data were collected at two time points, separated by four weeks. At Time 1 (T1), participants completed measures of abusive supervision of a coworker, deep-level similarity, perceived supervisor support, and moral attentiveness, along with their demographic information. At Time 2 (T2), participants rated their helping behaviors toward coworkers. Each questionnaire was assigned a unique code known only to the participant, allowing us to match responses across waves while maintaining anonymity. Participants sealed their completed questionnaires in envelopes and placed them into collection boxes located in the HR offices.

A total of 595 questionnaires were distributed at T1, and 530 were returned (response rate = 89%). After removing incomplete or unmatched responses, 500 valid paired surveys were retained (effective response rate = 84%). Among 500 participants, 47% work in food and beverage department, 32% work in front desk department, and 19.4% work in housekeeping department. In terms of sex, 43.2 were men and 56.8 were women. Their average age was 27.12 (SD = 7.32), average organizational tenure was 40.4 months (SD = 45.25), and average work hours per day was 8.5 (SD = 0.71).

### Measures

All the measurements were conducted in Chinese, following the standard translation-back-translation procedure (Brislin, 1970) to ensure the meaning equivalence across cultures.

Given that the present study aimed to investigate the reactions of the witness of abusive supervision of coworker, prior to completing the survey items, each participant was asked to recall the details regarding to a time when a coworker was treated in an impolite, unfriendly, or a rude manner by their immediate supervisor. Participants were further asked to provide the initial of that coworker. When participants were rating the survey items focusing on the abused coworker, they were required to refer to the situation and coworker identified in the situation they recalled. This recalling method has been used by (Mitchell et al., 2015) when they measured the emotional reactions of the witness of abusive supervision of coworker. We acknowledge that this recall-based procedure may be influenced by characteristics of the recalled episode (e.g., emotional intensity, victim–witness relationship closeness, and temporal proximity), which may introduce variability in participants’ reports. To reduce ambiguity, participants identified the coworker (by providing initials) and were instructed to answer all coworker-referenced items based on the same recalled incident and person.

#### Abusive supervision of coworker

We measured the abusive supervision of coworker using the 15-itme scale by Tepper (2000). The employees were asked to indicate how often they witness that their immediate supervisors engaged in the following listed behaviors toward the coworker they identified, on a 5-point Liker scale ranging from 1, never, to 5, very often. A sample item is “My supervisor ridiculous my coworker”. The Cronbach’s *α* for this scale is 0.94.

#### Deep-level similarity with coworker

A 3-item scale developed by Netemeyer et al. (2012) was adopted to measure the focal employee’s perceived similarity with the abused coworker. Since the original scale was designed to measure the extent of customer perceived similarity with the served employee, we changed the target from “employee” to “that coworker”. A sample item is “All things considered (values, personality traits, opinions, and preferences), I feel I am very similar to that coworker”. The employees were asked to evaluate each item on a 5-point Liker scale ranging from 1, strongly disagree, to 5, strongly agree. The Cronbach’s α for this scale is 0.89.

#### Perceived supervisor support

Perceived supervisor support was measured with 6 high loading items from the Survey of Perceived of Organizational Support (Eisenberger et al., 1986). This short scale has been examined with the good reliability and validity by previous research (Eisenberger et al., 2001). However, to assess the perceived supervisor support, we followed the (Eisenberger et al., 2002) modification procedure to replace the word of “organization” with “supervisor”. A sample item is “My supervisor is willing to help me if I need a special favor”. Employee were asked to evaluate each item on a 5-point Liker scale ranging from 1, strongly disagree, to 5, strongly agree. The Cronbach’s α for this scale is 0.80.

#### Moral attentiveness

We used 12 items developed by Reynolds (2008) to measure focal employee’s moral attentiveness. Employees were required to assess the extent to which they chronically consider the morality in their experiences on a 5-point Likert scale ranging from 1, strongly disagree, to 5, strongly agree. A sample is “In a typical day, I face several ethical dilemmas”. The Cronbach’s α for this scale is 0.88.

#### Helping behaviors toward the coworker

We used a 8-item scale of organizational citizenship behaviors directed to individual by Lee and Allen (2002). Employee were asked to indicate on average how often they provide the helps to the identified coworker on a 5-point Likert scale ranging from 1, never, to 5, very often. The Cronbach’s α for this scale is 0.88.

## Results

### Preliminary analysis

Table 1 showed the means, standard deviations, intercorrelations, and scale reliabilities. Prior to the hypothesis testing, we conducted the confirmatory factor analysis for the variables in our research model. First of all, to maintain the appropriate ratio of indicators and sample, we created parcels for all of study variables (Hagtvet and Nasser, 2004). To be specific, we randomly created 5 parcels cross 15 items measure of abusive supervision of coworker, 3 parcels cross 8 items measure of perceived supervisor support, 6 parcels cross 12 items measure of moral attentiveness, and 4 parcels cross 8 items measure of helping behaviors toward coworker. Then, we tested a model consisting of 5 study variables and results showed this hypothesized 5-factor fitted the data well (χ^2^ = 809.99, *df* = 179, CFI = 0.90, TLI = 0.88, RMSEA = 0.08, SRMR = 0.06). We further compared the hypothesized 5-facor model with two alternative models (i.e., 3-factor model and 1-factor model). The results of chi-square test indicated the hypothesized 5-factor model significantly had a better fit than 3-factor model (Δχ^2^ = 1334.63, Δ*df* = 7, *p* < 0.05) and 1-factor model (Δχ^2^ = 3591.28, Δ*df* = 10, *p* < 0.05). We further conducted average variance extracted (AVE) and composite reliability (CR) for each construct (see Table 2). Results showed that all the values of AVE and CR were adequate and above the recommended levels. The square root of AVE for each construct was greater than the correlation coefficients involving that construct. Taken together, these results demonstrated the convergent validity and discriminant validity of our measures.

**Table 1:** Descriptive statistics and correlation coefficients among the study variables.

Variables	Mean	Standard deviation	1	2	3	4	5
1. Abusive supervision of coworker	1.71	0.60	(0.94)				
2. Deep-level similarity with coworker	2.64	0.83	0.21**	(0.89)			
3. Perceived supervisor support	3.59	0.67	−0.25**	0.12**	(0.80)		
4. Moral attentiveness	3.04	0.64	0.06	0.30**	0.21**	(0.88)	
5. Helping behaviors toward the coworker	3.38	0.71	−0.05	0.29**	0.40**	0.21**	(0.88)

*n* = 500, **p < 0.01, *p < 0.01. Cronbach’s αs were reported in the parenthesis along the diagonal.

**Table 2:** Results of confirmatory factor analysis.

Measurement models	χ^2^	*df*	Δ χ^2^	Δ*df*	CFI	TLI	RMSEA	SRMR
Hypothesized 5-factor model	809.99	179	-	-	0.90	0.88	0.08	0.06
Alternative 3-factor model	2144.62	186	1334.63*	7	0.69	0.65	0.15	0.13
Alternative 1-factor model	4401.27	189	3591.28*	10	0.33	0.25	0.21	0.22

*n* = 500, *p < 0.05. Alternative 3-factor model: The measurement items of moderators (similarity, perceived supervisor support, and moral attentiveness) were merged into one factor, and items of abusive supervision of coworker and helping behaviors were loaded on their intended factors, respectively. Alternative 1-factor model: All the measurement items were merged into one general factor. CFI, comparative-fit index; TLI, Tucker-Lewis Index; RMSEA, root mean square error of approximation; SRMR, standardized root mean square residual.

### Hypothesis testing

We employed Mplus 7.2 (Muthén and Muthén, 2017) to test all the hypotheses and results were reported in Table 3. Hypothesis 1 proposed the moderating effect of similarity with the abused coworker in the relationship between abusive supervision of coworker and helping behaviors toward coworker. As prediction, the interaction term between abusive supervision of coworker and similarity with coworker was significantly related to helping behaviors toward coworker (*estimate* = 0.11, *p* < 0.05). Simple slope test helps us understand the pattern of interaction. In particular, abusive supervision of coworker was found to negatively relate to helping behaviors toward coworker when the similarity with coworker is low (−1 standard deviation, *simple slope estimate* = −0.17, *p* < 0.05), but the relationship becomes nonsignificant when similarity with coworker is high (+1 standard deviation, *simple slope estimate* = 0.01, *n.s.*). Hence, Hypothesis 1 was supported.

**Table 3:** Tests of discriminant and convergent validities of study variables.

Items	Parcel	Standardized factor loading	AVE	CR
Abusive supervision of coworker			0.72	0.93
1. I have witnessed that my supervisor ridicules this coworker.	Parceled item 1: Original item 1, 2, and 3 are parceled	0.84**		
2. I have witnessed that my supervisor tells this coworker’s thoughts or feelings are stupid.				
3. I have witnessed that my supervisor gives this coworker the silent treatment.				
4. I have witnessed that my supervisor puts this coworker down in front of others.	Parceled item 2: Original item 4, 5, and 6 are parceled	0.87**		
5. I have witnessed that my supervisor invades this coworker’s privacy.				
6. I have witnessed that my supervisor reminds this coworker of his/her past mistakes and failures.				
7. I have witnessed that my supervisor does not give this coworker’s credit for jobs requiring a lot effort.	Parceled item 3: Original item 7, 8, and 9 are parceled	0.88**		
8. I have witnessed that my supervisor blames this coworker to save himself/herself embarrassment.				
9. I have witnessed that my supervisor breaks promises he/she makes.				
10. I have witnessed that my supervisor expresses anger at this coworker when he/she is mad for another reason.	Parceled item 4: Original item 10, 11, and 12 are parceled	0.87**		
11. I have witnessed that my supervisor makes negative comments about this coworker to others.				
12. I have witnessed that my supervisor is rude to this coworker.				
13. I have witnessed that my supervisor does not allow this coworker to interact with other coworkers.	Parceled item 5: Original item 13, 14, and 15 are parceled	0.77**		
14. I have witnessed that my supervisor tells this coworker he/she is incompetent.				
15. I have witnessed that my supervisor lies to this coworker.				
Deep-level similarity with coworker			0.73	0.89
1. I feel I am very similar to this coworker.	Item 1: no parceling	0.84**		
2. I feel that I have a lot in common with this coworker.	Item 2: no parceling	0.92**		
3. All the things considered (values, personality traits, opinions, preferences), I feel I am very similar to this employee.	Item 3: no parceling	0.80**		
Perceived supervisor support			0.57	0.79
1. My supervisor takes pride in my accomplishments.	Parceled item 1: Original item 1 and 3 are parceled	0.58**		
2. My supervisor really cares about my well-being.				
3. My supervisor values my contribution to its well-being.	Parceled item 2: Original item 2 and 6 are parceled	0.80**		
4. My supervisor strongly considers my goals and values.				
5. My supervisor is willing to help me if I need a special favor.	Parceled item 3: Original item 4 and 5 are parceled	0.85**		
6. My supervisor shows little concern for me. (reversed item)				
Moral attentiveness			0.49^a^	0.85
1. In a typical day, I face several ethical dilemmas.	Parceled item 1: Original item 7 and 12 are parceled	0.44**		
2. I often have to choose between doing what’s right and doing something that’s wrong.				
3. I regularly face decision that have significant ethical implications.	Parceled item 2: Original item 1 and 2 are parceled	0.59**		
4. My life was been filled with one moral predicament after another.				
5. Many of the decision that I make have ethical dimensions to them.	Parceled item 3: Original item 3 and 4 are parceled	0.69**		
6. I regularly think about the ethical implications of my decisions.				
7. I think about the morality of my actions almost every day.	Parceled item 4: Original item 5 and 6 are parceled	0.79**		
8. I like to think about ethics.				
9. I frequently encounter ethical situations.	Parceled item 5: Original item 8 and 9 are parceled	0.79**		
10. I often find myself pondering about ethical issues.				
11. I often reflect on the moral aspect of my decision.	Parceled item 6: Original item 10 and 11 are parceled	0.84**		
12. I rarely face ethical dilemmas (reversed item).				
Helping behaviors toward the coworker			0.64	0.87
1. I help this coworker when he/she has been absent.		0.69**		
2. I am willing to give my time to help to this coworker when he/she has work-related problems.				
3. I adjust my work schedule to accommodate this coworker’s requests for time off.		0.81**		
4. I go out of the way to make this coworker feel welcome in the work group.				
5. I show genuine concern and courtesy toward this coworker, even under the most trying business or personal situations.		0.84**		
6. I give up time to help this coworker when he/she has work or nonwork problems.				
7. I assist this coworker with his/her duties.		0.84**		
8. I share personal property with others to help their work.				

^a^Fornell and Larcker (1981) note that AVE is a more conservative index, the convergent validity is evidenced even when value of AVE is lower than 0.5 but CR is adequate.

Hypothesis 2 proposed the moderating effect of perceived supervisor support in the relationship between abusive supervision of coworker and helping behaviors toward coworker. Table 3 also indicated the interaction term of abusive supervision of coworker and perceived supervisor support was significantly related to helping behaviors toward coworker (*estimate* = −0.23, *p* < 0.01). Moreover, the simple slope test showed the negative relationship existed between abusive supervision of coworker and helping behaviors coworker when perceived supervisor support is high (+1 standard deviation, *simple slope estimate* = −0.24, *p* < 0.01), but the relationship becomes nonsignificant when perceived supervisor support is low (−1 standard deviation, *simple slope estimate* = 0.08, *n.s.*). Hypothesis 2 was thus supported.

**Figure 2:**
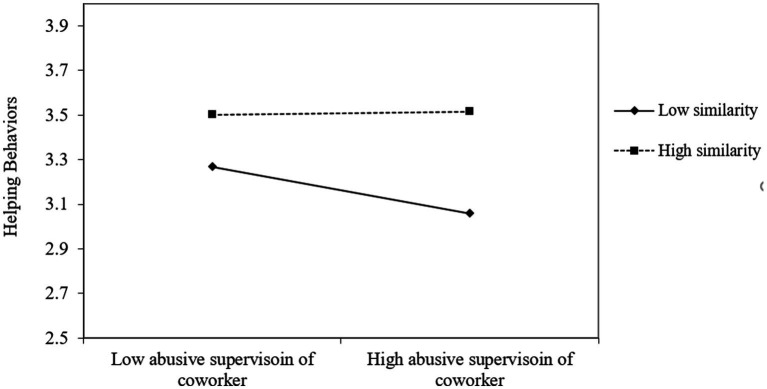
Interaction between abusive supervision of coworker and similarity on helping behavior.

**Figure 3:**
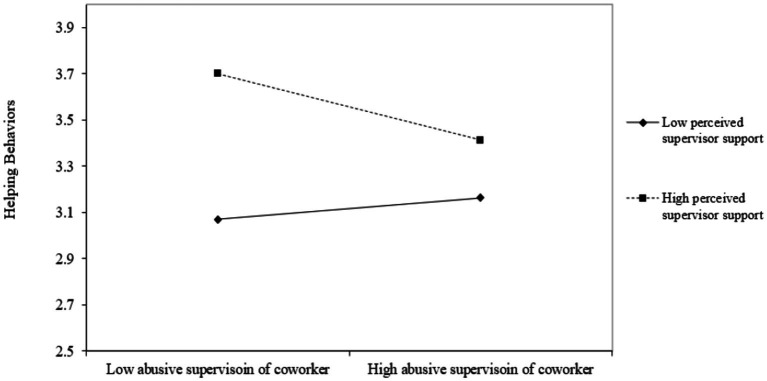
Interaction between abusive supervision of coworker and perceived supervisor support on helping behavior.

**Figure 4:**
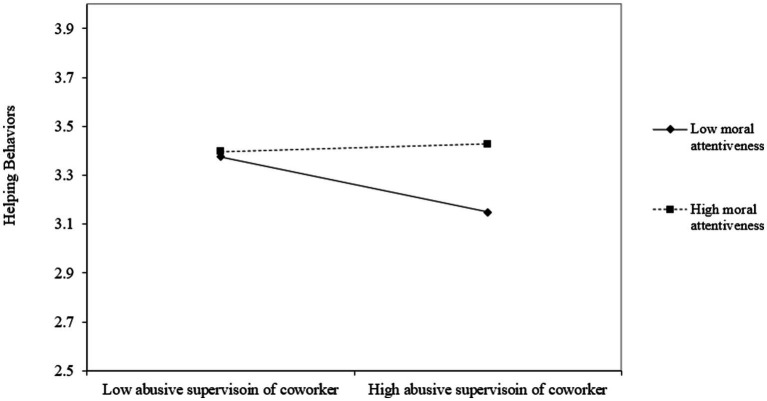
Interaction between abusive supervision and moral attentiveness on helping behavior.

Lastly, Hypothesis 3 proposed the moderating effect of moral attentiveness in the relationship between abusive supervision of coworker and helping behaviors toward coworker. Results showed the interaction term of abusive supervision of coworker and moral attentiveness was significantly related to helping behaviors toward the abused coworker (*estimate* = 0.17, *p* < 0.05). The simple slope test further revealed the negative relationship existed between abusive supervision of coworker and helping behaviors toward coworker when moral attentiveness was low (−1 standard deviation, *simple slope estimate* = −0.19, *p* < 0.05), but the relationship becomes nonsignificant when moral attentiveness was high (+1 standard deviation, *simple slope estimate* = 0.03, *n.s.*). All of these results provided the support for Hypothesis 3 (Figures 2–4; Table 4).

**Table 4:** Results of hypothesis testing in Mplus.

Paths	Estimates	Standard errors	*t*-values
Abusive supervision of coworker ➔ Helping behaviors toward the coworker	0.08	0.05	−1.61
Deep-level similarity with coworker ➔ Helping behaviors toward the coworker	0.21**	0.04	5.82
Abusive supervision of coworker*Deep-level similarity with coworker➔ Heling behaviors toward the coworker (H1)	0.11*	0.05	2.08
Perceived supervisor support ➔ Helping behaviors toward the coworker	0.33**	0.05	7.28
Abusive supervision of coworker*Perceived supervisor support ➔ Helping behaviors toward the coworker (H2)	−0.23**	0.08	−3.12
Moral attentiveness ➔ Helping behaviors toward the coworker	0.12*	0.05	2.40
Abusive supervision of coworker*Moral attentiveness ➔ Helping behaviors toward the coworker (H3)	0.17*	0.08	2.00

Simple slopes	Estimates	Standard errors	*t*-values
High deep-level similarity with coworker	0.01	0.06	0.17
Low deep-level similarity with coworker	−0.17*	0.07	−2.44
High perceived supervisor support	−0.24**	0.08	−3.03
Low perceived supervisor support	0.08	0.06	1.21
High moral attentiveness	0.03	0.07	0.38
Low moral attentiveness	−0.19*	0.08	−2.41

### Supplementary analysis on three-way interaction effects

Although not explicated hypothesized, it was expected that the relationship between abusive supervision of coworker and helping behaviors would be affected by these three moderators simultaneously, because these three moderators capture the different aspects of the perceptions and personal characteristic. Therefore, a series of supplementary analysis was conducted. Two significant three-way interactive effects were found. One is the interactive effects among the abusive supervision of coworker, similarity with coworker, and perceived supervisor support (estimate = 0.31, *p* < 0.01), and the other is the interactive effect among the abusive supervision of coworker, similarity with coworker, and moral attentiveness (*estimate* = −0.18, *p* < 0.01). To be specific, the significant negative relationships between abusive supervision of coworker and helping behaviors toward coworker was found only when (1) similarity with coworker was low and perceived supervisor support was high (*simple slope estimate* = −0.45, *p < 0*.01); and (2) both similarity with coworker and moral attentiveness were low (*simple slope estimate* = −0.35, *p < 0*.01). Consistent with our expectation and social identity theory, third party employee would be reluctant to provide the helps for the coworker when the supervisor identification (perceived supervisor support was high) was clearly prioritized over the coworker identification (similarity with coworker was low). Nevertheless, when they have the conflicting identification (both perceived supervisor support and similarity with coworker were low or high), they choose not to change their behaviors. Results also revealed that whether the third party employees who perceived the low similarity with coworker help their abused coworker or not depends on their moral attentiveness. Only when the third party employees were not properly aware of supervisor’s misconduct is a salient moral issue, they choose to distance from the abused coworker and reduce the helping behaviors. To summarize, this result further highlighted that similarity with coworker is a key driver of the helping behaviors.

## Discussion

### General discussion

Given the distinctive characteristics of the hotel industry, including its seasonal nature, high-intensity work rhythms (Chan et al., 2024), hierarchical organizational structures, frequent occurrences of abusive leadership, and high employee turnover rates (Cicerale, 2020; Park et al., 2020), abusive supervision is more prevalent in hospitality workplaces than in other industries (Wang et al., 2022). Given that this phenomenon is common in service industries (Yu et al., 2020), making it important to examine how such mistreatment shapes employee responses in these contexts.

The impact of abusive supervision on coworkers subjected to such behavior and third-party employee reactions have garnered significant attention from researchers in recent years. However, within the hotel management domain (Peltokorpi and Ramaswami, 2021; Xu et al., 2018; Yu et al., 2020), existing studies predominantly focus on the dyadic relationship between abusive supervisors and their directly affected subordinates, overlooking the broader and indirect impacts on third-party observers within the organization (Yu et al., 2022).

In previous research on abusive supervision and the three moderating variables, studies focusing on similarity have emphasized surface-level similarity between supervisors and subordinates, such as gender similarity and perceived demographic similarity (Bayhan Karapinar et al., 2024). Relatively few studies have integrated abusive supervision with deep-level similarity. This study, however, focuses on deep-level similarity and particularly explores the mechanisms of relationships among subordinates rather than limiting the scope to supervisor–subordinate relationships alone. We argue that deep-level similarity can profoundly influence interpersonal interactions and behaviors among subordinates. In addition, perceived supervisor support is less considered in the literature on abusive supervision. Previous research has generally focused separately on abusive supervision and supervisor support in hospitality industry. For example, Quratulain and Al-Hawari (2021) suggested that the effect of perceived supervisor support on employee adjustment is most significant among employees with high levels of cynicism when the diversity climate is high. Other studies have noted that abusive supervision was more strongly related to turnover for younger hospitality employees relative to older ones (Tews and Stafford, 2020). With respect to prior research on moral attentiveness, Pan et al. (2023) highlights that supervisors with high moral sensitivity who experience abusive supervision can play a key role in mitigating its spread. Afsar et al. (2019) found that abusive supervision has a weaker impact on the moral courage of subordinates with high moral attentiveness. This study thus expands and enriches the research on moral attentiveness and subordinate helping behaviors, providing new insights into these topics.

Overall speaking, based on social identity theory and deontic justice theory, we posited that the relationship between the abusive supervision of a coworker and helping behaviors toward that coworker was contingent upon the witness’s deep-level similarity, perceived supervisor support, and moral attentiveness. In previous research on deep-level similarity, most scholars have adopted the similarity-attraction paradigm. However, we chose social identity theory to elucidate the process of mutual identification between third-party employees and their coworkers. Research results have generally supported these assertions. When abusive supervision of a coworker and helping behaviors toward that coworker exist when deep-level similarity with the coworker is low, perceived supervisor support is high, and moral attentiveness is low. Interestingly, the results of the moderating effect were congruent across the three conditions. Specifically, we found that witnesses provide less help to their abused coworker under certain conditions, but we did not find that witnesses provide *more* help to their abused coworker under other conditions. This is reasonable and understandable because providing more help to the abused coworker would be seen as a direct offense against the abusive supervisor. It is not worth it for the witness to take risks to help a colleague. Therefore, witnesses may choose to maintain their interactions with the abused coworker, unchanged, as an indirect way to express their disagreement with the supervisor’s behavior.

Specifically, we found that a negative relationship between abusive supervision of a coworker and helping behaviors toward that coworker exists when deep-level similarity with the coworker is low, perceived supervisor support is high, and moral attentiveness is low. Interestingly, the results of the moderating effect were congruent across the three conditions. Specifically, we found that witnesses provide less help to their abused coworker under certain conditions, but we did not find that witnesses provide mor*e* help to their abused coworker under other conditions. This is reasonable and understandable because providing more help to the abused coworker would be seen as a direct offense against the abusive supervisor. It is not worth it for the witness to take risks to help a colleague. Therefore, witnesses may choose to maintain their interactions with the abused coworker, unchanged, as an indirect way to express their disagreement with the supervisor’s behavior.

### Theoretical implications

This research contributes to the literature on abusive supervision and helping behaviors in the following three aspects.

First, by including multiple moderators based on social identity theory and deontic justice theory, our research provides an integrated view of third-party employees’ reactions to the abusive supervision of a coworker. Although previous research has included social identity theory as a theoretical explanation for the relationship between abusive supervision and its consequences, most research has been limited to identifying with the work team or the organization and how abusive supervision harms the individual’s social identification at work. Instead of viewing identification as an outcome of the abusive supervision of a coworker, we build on previous work and shift the focus to how social identification intertwines with the abusive supervision of the coworker. Our research model shows the effects of individual identification with one or the other of the two parties in the event of abusive supervision of a coworker, i.e., the abusive supervisor or the abused coworker. To our knowledge, this study is the first to adopt social identity theory and to consider different identifications (with the victim or with the abuser) to examine the effect of abusive supervision of a coworker.

Second, our work further contributes to the literature on helping behaviors by including social relationships as the antecedents of helping behaviors. A review by Spitzmuller et al. (2008) mentioned that “attitudinal, dispositional, and motivational approaches to citizenship behavior fail to account for the social environment surrounding citizenship”. While prior studies have reported some primary findings regarding the effects of abusive supervision on the quality of the relationship with the abusive supervisor and helping behaviors toward the abusive supervisor (Xu et al., 2012), the findings of these studies are limited to a dyadic relationship. Therefore, we refined social relationships by developing a framework to examine how interactions with and perceptions of the perpetrator and victim influence employees’ helping behaviors within a group.

Third, recent hospitality management research has highlighted the importance of supportive behaviors (Quratulain and Al-Hawari, 2021) and abusive supervision (Dai et al., 2019), but little insight has been offered into their simultaneous effects (Chan et al., 2024). Additionally, the present study has shown that when supervisors display immoral behaviors and supportive behaviors simultaneously, employees may experience paradoxical and conflicting cognitions about those behaviors (Duffy et al., 2002). This conflicting understanding exacerbates undesirable outcomes (Chan et al., 2024). However, this assumption is not always justified. In some cases, the effect of a supervisor’s positive conduct is not offset by that of negative conduct or does not result in cognitive dissonance for the witness. Specifically, our research showed that when an employee is the recipient of the supervisor’s positive conduct but is not the target of the supervisor’s negative conduct, the supervisor’s supportive behaviors exert greater influence on the employee’s behavior. In addition, most prior research has examined these moderators in isolation. We integrate these three moderating variables to illustrate the dynamic behavioral processes among supervisors, subordinates, and third-party employees.

### Practical implications

Our research also has implications for the fields of leadership and team management. First, organizations should strengthen leadership training by creating a culture that encourages ethical behavior and open discussions about ethical issues (Treviño et al., 1998). To avoid this problem in the first place, organizations can rely on interviews or personality tests to identify individuals with high ethical standards, and then hire and train them for managerial positions, using similar techniques to assess whether leaders will effectively communicate the importance of ethics to employees (Al Halbusi et al., 2021). Second, to enhance employees’ moral attentiveness, regularly providing moral education and training can help employees identify and deal with ethical issues, thereby boosting their moral awareness (Weaver et al., 1999). Research has also shown that conducting various forms of ethics training, such as role playing, can significantly improve employees’ moral awareness and moral decision-making ability (Warren et al., 2014). We suggest that ethics training programs can encourage employees to actively discuss ethical and moral issues with colleagues can also help to enhance moral sensitivity. Recognizing and rewarding employees who display ethical behavior can reinforce the importance of such behavior.

Third, we recommend encouraging open, honest and mutually respectful communication among employees. Training courses on communication skills can help employees learn how to express themselves clearly and listen to others’ opinions (DeKay, 2012). Team-building activities can be used to break down communication barriers among colleagues, establish trust and rapport between team members, and foster closer and more harmonious relationships. It is important for organizations to let employees know about the available resources, whether instrumental support or emotional support, that they can access when facing challenges in the workplace.

### Limitations and directions for future research

First, this study employs a cross-sectional research design, which limits the researcher’s ability to infer causal relationships among the variables under investigation (Shadish et al., 2002). Future studies are suggested to adopt the experimental methods to confirm the casual relationships among the study variables or use mixed-methods approach to design research to provide a more robust result. In addition, although we used a time-lagged design to reduce common method variance, the key variables were still measured via self-reports, which may be subject to social desirability concerns. For example, participants may be inclined to portray themselves as more supportive or morally appropriate when reporting helping behaviors and related perceptions. Future research may further mitigate this concern by incorporating supervisor- or peer-rated helping behaviors, behavioral indicators, or multi-source designs.

Second, this study used deep-level similarity and perceived supervisor support as proxy measures to represent the extent of identification with coworker and supervisor. However, concepts of “identification with coworker” and “identification with supervisor” may best align with the principles of social identification theory. Future studies could directly measure these identification constructs to better capture the underlying social identity processes.

Third, although the present study did not assess witnesses’ own history of abusive supervision, which may be an important boundary condition in the bystander context. Witnesses with prior abuse experience may interpret coworker abusive supervision as more threatening, anticipate higher personal risk, or engage in greater avoidance, thereby shaping whether they provide help to the victim. Prior research suggests that personal experiences of abusive supervision may intensify negative reactions when individuals also witness abuse directed at others (Harris et al., 2013). Therefore, future research should explicitly measure and control for witnesses’ own abuse history and examine its potential interactive effects.

## Conclusion

This study advances understanding of coworker abusive supervision by examining when third-party employees choose to help or withhold discretionary assistance toward an abused coworker. Drawing on social identity theory and deontic justice theory, we show that witnesses’ helping is not a fixed response to observed mistreatment but depends on boundary conditions reflecting victim-related, perpetrator-related, and witness-related considerations. Specifically, the negative association between coworker abusive supervision and helping behaviors emerges primarily when witnesses perceive low deep-level similarity with the victim, high perceived supervisor support, and low moral attentiveness, whereas in other conditions helping tends to remain unchanged.

These findings contribute to the abusive supervision and workplace helping literatures by highlighting a tripartite perspective (perpetrator–victim–witness) and clarifying when supportive responses are constrained in coworker abuse episodes. Practically, the results underscore the importance of strengthening ethical leadership practices and reducing supervisors’ abusive conduct, while also promoting ethical awareness and supportive coworker climates to protect service functioning in hospitality organizations where cooperation and coordination are critical.

## Data Availability

The original contributions presented in the study are included in the article/supplementary material, further inquiries can be directed to the corresponding author.
